# Real-Time Task Assignment Approach Leveraging Reinforcement Learning with Evolution Strategies for Long-Term Latency Minimization in Fog Computing

**DOI:** 10.3390/s18092830

**Published:** 2018-08-27

**Authors:** Long Mai, Nhu-Ngoc Dao, Minho Park

**Affiliations:** 1 Department of Information Communication, Materials, and Chemistry Convergence, Soongsil University, Seoul 06978, Korea; longmaisg@ssu.ac.kr; 2 School of Computer Science and Engineering, Chung-Ang University, Seoul 06974, Korea; dnngoc@uclab.re.kr

**Keywords:** real-time task assignment, fog computing, reinforcement learning, evolution strategies, long-term latency minimization

## Abstract

The emerging fog computing technology is characterized by an ultralow latency response, which benefits a massive number of time-sensitive services and applications in the Internet of things (IoT) era. To this end, the fog computing infrastructure must minimize latencies for both service delivery and execution phases. While the transmission latency significantly depends on external factors (e.g., channel bandwidth, communication resources, and interferences), the computation latency can be considered as an internal issue that the fog computing infrastructure could actively self-handle. From this view point, we propose a reinforcement learning approach that utilizes the evolution strategies for real-time task assignment among fog servers to minimize the total computation latency during a long-term period. Experimental results demonstrate that the proposed approach reduces the latency by approximately 16.1% compared to the existing methods. Additionally, the proposed learning algorithm has low computational complexity and an effectively parallel operation; therefore, it is especially appropriate to be implemented in modern heterogeneous computing platforms.

## 1. Introduction

Fog computing was developed to act as an intermediate between a remote cloud computing environment and Internet of Things (IoT) devices. It is a novel architecture that extends the cloud to the edge of the network [1,2]. In fog computing, latency-sensitive tasks can be executed at the fog servers, near the devices, while delay-tolerant and computationally intensive applications can be offloaded to the cloud. Fog computing also provides additional advantages such as the ability of processing applications at specific locations. Owing to these advantages, the fog computing infrastructure is increasingly utilized for handling real-time IoT services and applications [3,4,5].

While fog computing deployment provides substantial benefit over cloud computing, it exposes a critical challenge in terms of task assignment problem [6,7]. If tasks are not assigned to suitable servers, some servers may suffer from a burden in processing while others with rich resources relax [8]. Particularly, the imbalance in resource utilization is heightened in scenarios where a large number of IoT devices are present. Consequently, efficient task assignment techniques in real-time are inevitable for fog networks, especially over a long-term period to achieve system stability.

To overcome the aforementioned issues, we proposed a real-time task assignment approach, which leverages reinforcement learning (RL) [9,10,11] with evolution strategies (ES) training method [12,13] for long-term latency minimization in fog computing. In this approach, a central scheduler that performs task assignment among fog servers considers the fog computing infrastructure as a trainable neural network (NN) [14,15]. In the NN, fog computing resources, remaining tasks in the buffers of fog servers, and demand of the offloaded task make up various states of the system. The number of system states is extremely huge; therefore, the ES algorithm has been utilized for learning operations for obtaining a fast optimization of long-term latency minimization as a training reward.

We experiment task assignment in the fog computing of a factory, where IoT devices are frequent but have noise interference. The system includes 200 IoT devices and 10 fog servers with different capabilities of task processing. Although the complexity of real-time task assignment problem is high, the proposed reinforcement learning model with evolution strategies algorithm reaches the objective of optimizing long-term latency and has 16.1% higher reward than the greedy method, which is the baseline in real-time task assignment. The contributions of this study are as follows.

1. We propose a reinforcement learning model for the real-time task assignment in fog networks with the objective of minimizing long-term latency. The method for crafting states of the system is novel and is an important contribution to the success of the model.
2. We propose the evolution strategies as a learning method for the reinforcement learning model for optimizing the server selection function, i.e., the trainable neural network. The algorithm has low computational complexity and simplicity in implementation. Additionally, the algorithm is remarkably parallel due to the independence in evaluation of its children. Therefore, it is suitable for modern computers with parallel CPUs.
3. We prove by comprehensive experiments that the proposed model is scalable when the system escalates the number of IoT devices or the number of fog servers. The model attains 15.3% higher reward than the greedy method in a system with 100 IoT devices and five fog servers; and 16.1% with 200 IoT devices and 10 fog servers.

The rest of the paper is organized as follows. Section 2 surveys work related to the fog computing paradigm and real-time task assignment problem. In Section 3, we propose the reinforcement learning model and how to craft the states of the system. Section 4 introduces the evolution strategies as a learning algorithm for the proposed model. Section 5 shows the experiment results for proving the efficiency of the proposed model and exploring the effectiveness of the parameters of the model. Section 6 concludes the study.

## 2. Related Work

Literature reviews [16,17] revealed a great contribution of research communities for improving fog computing performances in terms of latency, energy consumption, resource utilization, service availability, their variants, and hybrid solutions [2,18,19,20,21,22]. In the latency minimization objective, the state-of-the-art solutions, however, mainly considered the fog computing on the basis of time intervals. Meanwhile, the effective real-time operations that require immediate reaction immediately after the tasks arrive at the fog computing infrastructure have not yet been significantly taken into account.

For instance, Chamola et al. [23] proposed a load balancing scheme among fog servers for minimizing the total computation latency in the entire system during an observed period. The developed latency optimization function was relaxed to be a convex problem for making it resolvable. Although a total latency reduction is achieved, this work did not consider the offloaded task complexity, which has significant effects on the fog computing performance. Moreover, the algorithmic complexity restricts the scheme to be applied to a large-scale environment. In [24], a pattern-identified online task scheduling (PIOTS) mechanism has been proposed for industrial IoT services in multitier fog computing systems. The PIOTS mechanism learned task arrival based on historial task data using the self-organizing map (SOM) technique and further applied the Hungarian assignment method for the identified task patterns. Based on that, incoming tasks arriving at the fog computing system are matched into these patterns and applied to the appropriate assignment policies for obtaining the total system latency minimization. However, the dimension of the SOM is constant; therefore, the PIOTS mechanism does not flexibly adapt to time-varying task arrivals. In [25], Ali et al. considered the latency-aware cloudlet selection in a fog network as a many-to-one matching game, where the IoT devices and cloudlets rank each other for latency minimization. A distributed and self-organizing method was proposed for solving the matching game to obtain the objective function. However, the latency in this work considered the system operation per timeslot, which is not truly a real-time environment. On the contrary, Dao et al. [26] proposed an adaptive resource balancing (ARB) scheme by migrating user services among fog servers (located at fog radio access nodes). The ARB orchestrates workload in the entire system using the backpressure algorithm so as to minimize the computation latency (i.e., serviceability maximization). Similar to the mentioned studies, the ARB scheme has limitations due to its timeslot-based optimization approach. The optimization for the time intervals requires that tasks generated by IoT devices have to be divided into fragments with a fixed size to be uploaded in time intervals, which is not a trivial process [1]. Additionally, the approach assumes that the servers have to complete the assignments and their buffers are cleared after each time period, which is not practical.

**Figure 1:**
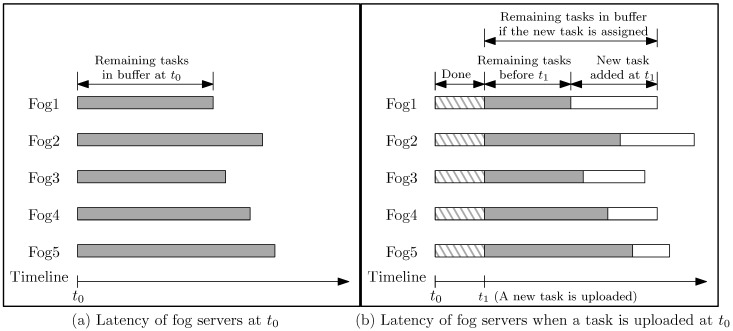
Real-time task assignment considering buffer status of fog servers.

By contrast, the greedy method is commonly used while considering real-time processing. The function of this method is illustrated in Figure 1. At $t_{0}$ (Figure 1a), there are some servers in processing. When a task is uploaded at $t_{1}$ (Figure 1b), some tasks have been done and there are tasks remaining from the previous task list in the buffers. This method determines the demand of a task and the status of buffers for selecting a server with the objective of minimizing latency at that moment. In Figure 1, server Fog3 has the lowest latency in processing the remaining tasks and the new task, e.g., the new task is assigned to the server. Although the greedy method is more realistic compared to time interval approach, it is not functional toward long-term latency optimization. Section 3.1 shows an extreme case that the greedy method does not achieve the minimal latency in the long-term, which proves that the method is not sufficient for real-time task assignment.

In summary, almost all related works lack the truly real-time consideration and a flexible task arrival adaptation. Therefore, an effective solution, which aims at resolving these problems, is crucial for the fog computing system.

## 3. System Model

### 3.1. Real-Time Task Assignment Problem

In the real-time task assignment problem, we define a system to be a fog network that includes IoT devices that generate tasks, fog servers with various capabilities in processing a task, and a task assignment module that chooses servers where the tasks are executed. The tasks are uploaded in real-time in the system, which means that a time interval between two consecutive tasks is in the range [0,∞]. Each fog server has a buffer for remaining tasks with unlimited capacity. For the sake of simplicity, we assume that an IoT device always generates tasks with the same size and complexity. In this study, we consider a scenario in a factory where tasks uploaded by IoT devices are frequent but occasionally have an interference of random noise. As there are many devices that continuously upload tasks, traffic reaching to fog servers is extremely noisy and the complexity of the problem is high. Table 1 lists the explanation of terms used in the study.

It is observed that the total latency consists of propagation, execution, and buffering. To adapt to various networking environments, we consider the IoT task arrival as a random and independent process on the communication channels between the IoT devices and fog servers. In other words, the propagation latency is omitted in the scope of this study. Let $\langle s_{i},c_{i},\tau_{i}\rangle$ denote a three-dimensional characteristic vector of the *i*-th task, where $s_{i}$, $c_{i}$, and $\tau_{i}$ are the size, complexity, and latency threshold of the task, respectively. In addition, let $f_{j}$ and $b_{j}$ denote the CPU frequency and current buffer size of the *j*-th fog server, respectively. Assume that the *i*-th task is assigned to the *j*-th fog server, and then the latency of *i*-th task is given by

(1) $$L_{ij}=\frac{s_{i}c_{i}+b_{j}}{f_{j}}.$$

Based on the system described above, we define a real-time task assignment problem as selecting a fog server for assigning a task to minimize the computation latency of the system during its operational time, which is referred to as long-term latency optimization. To mathematically express this problem, let $x_{ij}$ denote the case when the *i*-th task is assigned to the *j*-th fog server. The latency minimization function at timeslot *t* is defined by

(2) $$\begin{array}{cc} P(t)≜\min & \sum_{i=1}^{\Omega (t)}\sum_{j=1}^{\Psi }x_{ij}L_{ij} \end{array}$$

(3) $$\begin{array}{cc} s.t.\; & \sum_{i=1}^{\Omega (t)}x_{ij}=1,\;\forall j\in \Psi , \end{array}$$

(4) $$\begin{array}{c} L_{ij}\leq \tau_{i},\;\forall j\in \Psi , \end{array}$$

(5) $$\begin{array}{c} x_{ij}\in \left\{0,1\right\},\;\forall i\in \Omega \left(t\right),\;\forall j\in \Psi , \end{array}$$

where Ω(t) and Ψ are the sets of the IoT tasks and fog servers, respectively. Therefore, the long-term latency minimization function (F) is given by

(6) $$\left(F\right)\;\lim_{t\to \infty }\frac{1}{t}\sum_{i=1}^{t}P\left(i\right).$$

**Figure 2:**
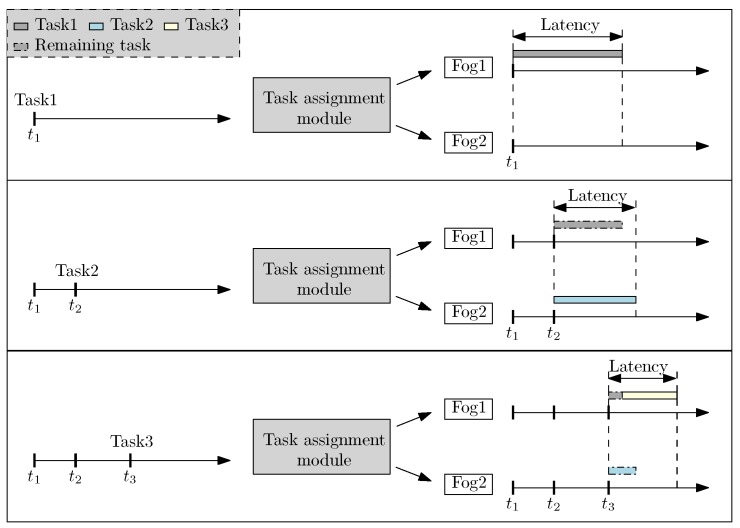
Task assignment overview.

To clearly demonstrate the problem, we consider an extreme case in Figure 2 when there are only two fog servers: Fog1 and Fog2, and three tasks are uploaded in order. In addition, the buffer in fog servers are assumed to operate with first-come-first-serve (FIFO) policy. The heart of the system is a task assignment module, a.k.a task scheduler, for deciding servers where the tasks are to be executed. At $t_{1}$, Task1 is uploaded and assigned to Fog1. The dark bar represents a time span needed by Fog1 to process Task1. Computational latency of the system is considered to be the maximum latency among fog servers to completely process the uploaded tasks. At $t_{2}$, Task2 is uploaded and the module chooses Fog2 for processing the task. The module makes decision by using the greedy method, which minimizes the latency of the system at the moment when a task is uploaded. It is worth noting that at $t_{2}$, a part of Task1 is completed by Fog1 and the remaining task is in the buffer of the server; this is indicated by a bar with a dashed dotted choke. Adopting the greedy policy, Task3 is uploaded and assigned to Fog1 since the current buffer of Fog2 is larger than Fog1’s at $t_{3}$. Consequently, the buffer of Fog1 contains both the remaining tasks of Task1 and the new task of Task3, whereas the buffer of Task2 contains the remaining task of Task2. At that moment, system latency is caused by Fog1.

In the extreme case mentioned above, we used the greedy method for task assignment. We now present an example in Table 2a for explaining the details about the method. As shown in the table, expected latency is the time that a fog server needs to solve a task; Fog(n) latency is the time that the server Fog(n) needs to process all remaining tasks in its buffer. The capability of Fog1 is 2 GHz, i.e., the server has the ability to process two gigacycles of tasks in a second. Right before the moment 0 ms, all buffers are empty and the system latency is 0. Task1 is uploaded at 0-ms time point. There are 1 Mbits × 10 cycles/bit = 10 Megacycles needed to process the task. Therefore, Fog1 and Fog2 need 5 ms and 10 ms for the task, respectively. In the real-time task assignment, the greedy method chooses a fog server for the task assignment with an objective of minimizing system latency at the moment the task is uploaded. Thereby, Task1 is assigned to Fog1. At this moment, Fog1 and Fog2 need 5 ms and 0 ms, respectively, to finish all the tasks in their buffers. Here, the system latency is 5 ms.

At 2-ms time point, Fog1 needs 3 ms to finish the remaining tasks in its buffer. Task2, which needs 3.5 ms and 7 ms by Fog1 and Fog2 to process it, respectively is consecutively uploaded. If Task2 is assigned to Fog1, the server needs 3 ms + 3.5 ms, i.e., 6.5 ms to finish the tasks. In case Task2 is assigned to Fog2, the server needs 7 ms to finish the tasks. Following the greedy method, Task2 is assigned to Fog1, and its latency is 6.5 ms. Task3 is uploaded right after that moment; it needs 4 ms by Fog1 and 8 ms by Fog2 to process it. In case Task3 is assigned to Fog1, the latency of Fog1 is 6.5 ms + 4 ms, i.e., 10.5 ms. However, in case Task3 is assigned to Fog3, then the latency of Fog2 is 8 ms. Therefore, Task3 is assigned to Fog2 and the system latency becomes 8 ms, corresponding to the latency of Fog2.

However, the greedy method is not suitable for long-term latency optimization. In the example above, if focusing on long-term latency, we can propose a better solution to the task assignment problem as shown in Table 2b. In the table, when Task2 is assigned to Fog2, system latency is 7 ms, which is an increase from the latency of 6.5 ms in case of the greedy method. However, Task3 is assigned to Fog1; this causes the latency of the system to be 7 ms. As a result of the change in task assignment, system latency right after the moment 2 ms reduces (7 ms compared to 8 ms when we apply the greedy method). Intuitively, given the state of a system, which includes task demand (size and complexity) and status of server buffers, the action of assigning a task to a server changes the state of the system at that moment, and a reward is returned, e.g., an inverse of latency. Since the tasks that are uploaded are not totally random but frequently with noise, there should also exist a reward pattern when the determined system states are given. Therefore, in this study, we utilize reinforcement learning for exploiting the pattern of the pair state-reward to minimize the latency of the system in the real-time task assignment.

### 3.2. Reinforcement Learning Model

Reinforcement learning (RL) is a class of machine learning, besides supervised learning and unsupervised learning [15,27]. The objective of an RL problem is automation and control of a system for adapting to an unknown environment [9,28,29,30]. In our problem, the training environment is the system consisting of fog servers and their buffers. It is worth noting that the environment is consistent, e.g., for each condition, it expresses a unique state. In other words, a state is representative of the environment at the moment we observe it. At the center of the RL model is the action selection function, briefly, the action function (i.e., task assignment module in the central scheduler). The function selects actions based on the states of the system. Each time the system conducts an action, the condition of the environment changes and a new state is expressed. A reward is also assigned to the system for indicating its adaptation. Thereby, the objective of the model is to maximize the rewards received, e.g., maximize the adaptation of the system to the environment. In turn, the action function has to reinforce itself to enhance its ability in choosing actions efficiently by harnessing the rewards. When a new state is expressed, the learning loop continues and the action selection function is continuously reinforced. In the proposed model, the action selection function is a trainable neural network [14] and the learning rule of the function follows the algorithm mentioned in Section 4.

In this paper, we aim to construct a novel approach to craft states of the system in the problem as follows. Given a system presented in Section 3.1, at a moment that a task is uploaded (but still not yet assigned), there are values that can express the state of the system: (1) demand (e.g., size and complexity) of the task, (2) remaining tasks in the buffers of the servers, (3) time span from the last moment of uploading the task to the current moment, (4) a chain of demands from the last tasks. As the third factor is affected by noise and the last factor could cause a burden to the computers used in training the model, only the first and second factors are chosen to define the state. We craft a state of the system from the two factors as follows. At the moment when a task is uploaded, there are some remaining tasks in the buffers of the servers. First, we measure time durations that the servers have to complete the remaining tasks, e.g., computation latency of the servers, and present the values in a vector of size $\left[n\times 1\right]$. Next, we calculate time durations that the servers will spend if the arrived task is assigned, then the values are stored in another vector of the same size $\left[n\times 1\right]$. Combining the above-mentioned two vectors, we obtain a vector of size $\left[2n\times 1\right]$, which represents the state of the system at a given moment.

**Figure 3:**
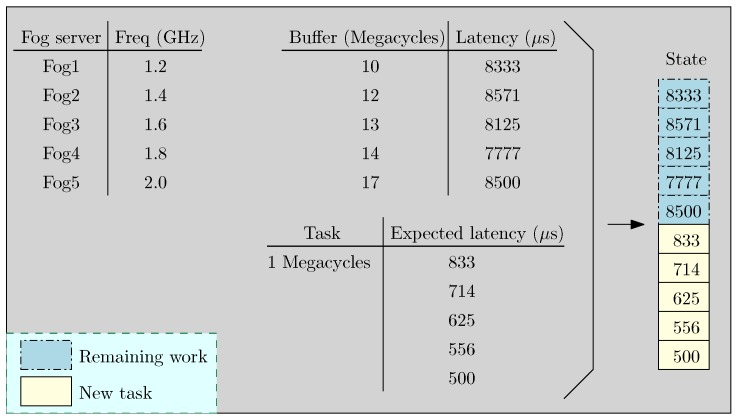
An example of a state.

Figure 3 illustrates a state in the experiments discussed in Section 5. In the figure, there are three tables. The first table presents five fog servers and their frequencies. The second table lists the remaining tasks in the buffers of the servers at the moment a task is uploaded. We measure the computation latency of the servers for completing the remaining tasks in microseconds (μs). The task requires 1 megacycle for its completion, which leads to a variation in the expected latency among the servers. Based on the latency in processing the remaining tasks, and the expected latency in completion of the task, we craft the state of the system at this moment as a vector of size $\left[10\times 1\right]$.

### 3.3. Action Selection Function

Action selection function in the RL model is a trainable machine learning (ML) function that reinforces its ability in action selection through rewards. Among various ML functions that are applied to the RL model, the neural network (NN) is the most popular [13,14,15]. Since the NN is a universal approximation function, it can fit well to various types of RL problems [31]. Additionally, a combination of the RL and NN has also shown the ability to surpass human level in many applications such as the game of Go [32]. Therefore, NN is chosen to be the action selection function in the proposed RL model.

**Figure 4:**
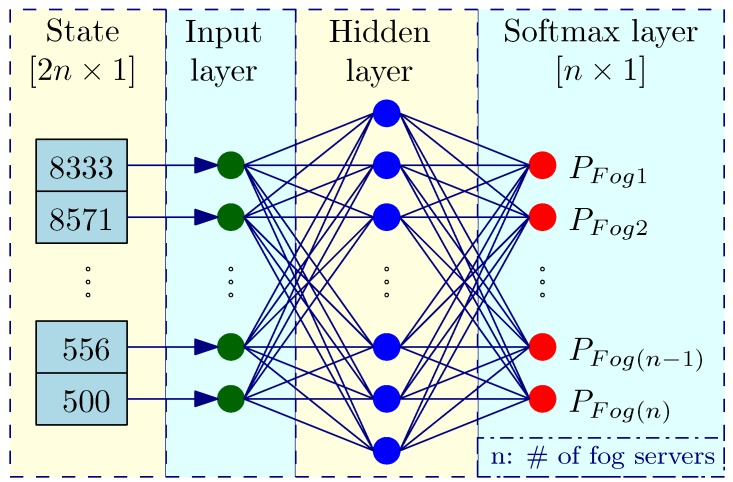
Neural network in reinforcement learning.

An example of the NN is demonstrated in Figure 4, which has three layers. The state of the system is an input to the NN. Since a state has size $\left[2n\times 1\right]$, input layer of the NN also has 2n nodes, which are denoted as $x^{\left(i\right)},i=\left\{1,\ldots ,n\right\}$. All nodes in the input layer connect to all nodes in the hidden layer. Given that the hidden layer has *m* nodes, we denote them as $h^{\left(j\right)},j=\left\{1,\ldots ,m\right\}$. Therefore, there are $\left[m\times n\right]$ connections between the input and the hidden layers. Given that each connection has a weight, there exists a matrix $W^{\left(1\right)}$ with the capacity of storing all weights. Weight $W_{i,j}^{\left(1\right)}$ at row *i* and column *j* represents a connection between the two nodes $x^{\left(i\right)}$ and $h^{\left(j\right)}$. The value of a node $h^{\left(j\right)}$ in the hidden layer is a sum of all the products of weights and inputs.

(7) $$h^{j}=\sum_{i=1}^{2n}\left(w_{i,j}^{\left(1\right)}\times x^{\left(i\right)}\right)$$

The number of nodes in the hidden layer can affect the training process; therefore, it is carefully explored in the experiments in Section 5. All nodes in the hidden layer are connected to the softmax layer, which is also the output layer of the NN. Size of the output layer is $\left[n\times 1\right]$. A matrix W(2) which has size $\left[m\times n\right]$ stores all weights of connections between the two layers. Thereby, the value of a node ${\hat{y}}^{\left(k\right)}$ in the last layer is calculated as follows.

(8) $${\hat{y}}^{\left(k\right)}=\sum_{j=1}^{m}\left(w_{j,k}^{\left(2\right)}\times x^{\left(i\right)}\right)$$

After the values of all nodes are calculated, the probability that a fog server Fog(i) is selected, where i=1,⋯,n is derived by the softmax function as follows.

(9) $$P_{Fog(i)}=\frac{{\hat{y}}^{\left(i\right)}}{\sum_{k=1}^{n}{\hat{y}}^{\left(k\right)}}$$

The server that has the highest probability is chosen for assigning the uploaded task.

The NN is trained by updating its weight matrices, e.g., $W^{\left(1\right)}$ and $W^{\left(2\right)}$, to maximize the feedback from the environment. A popular method for updating the weight matrices is through backpropagation, which has proven effective in some RL applications such as the game of Go [14,33,34]. However, the method requires the feedback to be scaled to integer levels of {0, 1}, which is not efficient in this problem, where the latency is a floating value. Therefore, in this study, we choose a neural network evolution algorithm [12], which is a rival of backpropagation algorithm, for training the NN. The algorithm is successfully utilized in RL problems of OpenAI and UberLab [13,35].

## 4. Evolution Strategies

To train an ML model, we define an objective function for measuring how well the model is performing in a problem and optimize the ML model based on the function. Given the RL to solve the task assignment problem, our objective is choosing an action for minimizing the long-term latency of the system. However, the RL is defined to train for maximizing rewards from the system. Consequently, a reward is an inverse of the system latency. More precisely, a reward from the system after choosing an action a(t) is defined as follows.

(10) $$Reward=\frac{1}{L(t)},$$

where $L\left(t\right)=L\left(t-1\right)+L_{ij}\left(t\right)$, L(0)=0, and $L_{ij}\left(t\right)$ is the latency generated by the action a(t), which assigns the incoming task (*i-th* task) to the *j-th* fog server. It is seen that when t→∞, L(t)→∞ and the *Reward*→0. Coordinating with the function F in Equation (6), the objective of minimizing the long-term latency of the system can be relaxed into minimizing latency over *n* consecutive tasks, changes into the objective of maximizing the average of rewards over *n* recent actions. Therefore, the *Reward* function is given by

(11) $$Reward=\frac{1}{\sum_{k=t-n}^{t}L_{ij}\left(k\right)}$$

To optimize the RL model following the rewards, we update the NN to enhance the ability of the model in choosing actions for task assignments.

Backpropagation is the most popular algorithm for updating the NN. The algorithm calculates the derivatives of the objective function given by weights of the NN and updates the network toward maximizing the objective. However, in our problem, if we update the NN following the current action but not future actions, we cannot attain long-term optimization. Some backpropagation-based paradigms are proposed for optimizing the long-term reward (e.g., Deep Q-Learning [36]). However, in practice, such paradigms only work well if the reward received from the environment is either 1 or 0 (which means winning or losing the game). The following drawbacks hinder the algorithms’ success in the real-time task assignment problem since the reward from the environment (e.g., the inversed latency of the system) is arbitrarily floating values.

Neuroevolution (NE), i.e., neural network evolution, which is inspired by biological evolution, is another approach for training neural networks [12]. In nature, evolution begins when parents produce an offspring with random deviation. Among the children, those who fit the environment have better opportunity to survive and reproduce their genomes. As a result of the selection, the next generation enhances the fitness to the environment. The concept of NE is similar to evolution in nature. Given an NN, for each of its iterations, a new generation is produced from the NN, which includes derivations of the NN. The children that have the highest rewards are chosen and the NN is updated based on the rewards. The method to update the NN is conducted in evolution strategies (ES), which is the most well-known algorithm that applies the NE approach [13,35].

Algorithm 1 describes the process for updating the NN by ES. For each iteration, *m* children of the NN are produced by adding Gaussian noise to each weight in the network. Each child NN plays a role as task assignment module in the RL model with *n* consecutive tasks and receives an average reward over *n* actions. Since an average reward is the feedback of the system to the actions chosen by the child, it is also the fitness of the child to the environment. We calculate the mean reward of *m* children and differences of the rewards of children with the mean reward, which is also the gain of the children over the root network. If a child has a gain, it has better fitness than the root network and should be encouraged to contribute more to the next generation. Following that idea, the root NN is updated by adding weights of children to its weights toward the gain of the children.

(12) $$W_{j,k}^{\left(i\right)}=W_{j,k}^{\left(i\right)}+\eta \times \sum gain^{\left(h\right)}\times W_{j,k}^{\left(i)(h\right)},\;h=1,\cdots ,m,$$

where *h* and η are the number of children and the learning rate (how fast should we update the weights of the NN), respectively. It is worth noting that since the gain of a child is a difference between the mean reward of *m* children and its reward, the gain can be negative. In that case, Equation (12) discourages the child to contribute to the reproduction of the next generation.

**Algorithm 1** Reinforcement learning with evolution strategies.1: **Given**2:     Parent NN with weight matrix $W^{\left(i\right)}$
i=1,2
3:     number of children *m*4:     learning_rate η5: **Start**6:     **for** iteration in a predefined range **do**7:         **for**
*h* in range *m*
**do**8:            $Child^{\left(h\right)}=$ Parent NN + random noise($W^{\left(i)(h\right)}=W^{\left(i\right)}+noise$)9:            Evaluate $Child^{\left(h\right)}\to Reward^{\left(h\right)}$10:         Calculate Mean_reward11:         $Gain^{\left(h\right)}=Reward^{\left(h\right)}-Mean\_reward$
h=1,…,m
12:         Parent NN → Parent NN + $\eta \times \sum_{h=1}^{m}Gain^{\left(h\right)}\times Child^{\left(h\right)}$   
$W^{\left(i\right)}=\eta \times \sum Gain^{\left(h\right)}\times W^{\left(i)(h\right)}$
13:         Evaluate Parent NN14: **End**
**Return** the highest performing Parent NN

In summary, by adding random noise to the copies of the NN, the ES algorithm generates a population of networks in the nearby area of the NN. For each iteration, the NN moves toward the area that offers high rewards (positive gain) and avoids the area that offers negative gain. Over several iterations, the algorithm seeks the area that offers the best reward, e.g., the optimization of the RL model for the task assignment problem.

Since the ES algorithm seeks the gain in the nearby area during each iteration, it is important to control the deviation noise added to the children. If the area of a child is very near the root NN, the network may be stuck in the small area. However, if a child is too far from the root NN, we may skip the area that could be the solution to the problem. In the experiments in Section 5, the deviation of the children is searched by a practical method. It is worth noting that the ES algorithm does not depend on the derivative of the reward function, hence, it is not stuck in the local optima as the back-propagation algorithm which is based on gradients. On the other hand, each child functions independently from the others; therefore, the computation of the ES is parallel. This makes the ES algorithm work efficiently in a modern computer, which has many parallel CPUs, whereas the Deep RL models with backpropagation algorithm can only update in a single CPU environment.

## 5. Experiments

### 5.1. Experimental Setup

#### 5.1.1. Data Collection

We set up a real-time task assignment system for conducting 11 experiments. There are 100–200 IoT devices in each experiment. All devices are active with task uploading frequencies in the range [10,250] ms. For each device, the probability that a task is uploaded with abnormal frequency, i.e., the task is uploaded at a sooner or later time than expected time, is 5%. This abnormal task uploading represents a noise interference of the data. If a task is uploaded at a random time, it does not violate its predecessor or successor. For instance, if a random task is uploaded at $t_{1}$, then its predecessor and successor are uploaded at $t_{0}$ and $t_{2}$, respectively. $t_{1}$ guarantees that $(t_{1}-t_{0})>0$ and $(t_{2}-t_{1})>0$. With hundreds of IoT devices with 5% random and each device’s initials at different times, the number of possibilities of system states is immense.

For each device, the size and complexity of each uploaded task are similar and do not change during its lifetime. The sizes of tasks are in the range [1,100] kbits and their complexities are in the range [10,200] cycles/bit. Consequently, the requirement for a task ranges from 10,000 cycles to 10 Megacycles. Since we run the experiments with Python 3.6, the minimum time scale in the experiments is 1 μs. This means if the uploading times of two tasks are not the same, then their discrepancy is at least 1 μs. It is worth noting that the minimum timescale is practical, particularly in factories.

We collect task uploading in one hour and save the tasks in a dataset along the uploading time. The number of tasks uploaded is over 12 million. The average number of tasks uploaded in one second is 2700, and the average task rate per second (which is a sum of requirements of all tasks in one second) is 7.68 gigacycles.

#### 5.1.2. Fog Server and System Setup

In the experiments, five fog servers are utilized for task assignment. We define the capability of a server to be the number of cycles that the server can solve in one second. It is also the frequency of the server. For instance, if a server has the frequency of 1 GHz, then it has a capability of solving the tasks at a rate of 1 gigacycles per second. Total capability of all servers should be higher than the average task rate so that the servers can solve all the uploaded tasks. In the experiments, five servers are designed to have frequencies in the range {1.2,1.4,1.6,1.8,2.0} GHz; hence, the average task rate of 7.68 gigacycles is equal to 96% of the total capability of the servers. It is worth noting that the problem of fairly allocating resources between fog servers is out of the scope of the study.

The system is simulated using Python 3.6. In the experiments, we suppose that the buffers of the servers are unlimited. Computation latency, e.g., a time span for a server to execute remaining tasks in its buffer, has the minimum scale in μs, and follows the smallest scale of time in Python 3.6. It is worth noting that in the literature, we only consider computation latency in fog servers. Transmission delay and queue delay are not covers in the study. The simulation runs on a single computer with a CPU core i7-4770 3.4 GHz. The computer has 16 GB memory and no GPU is used in the simulation.

### 5.2. Experimental Method

**Figure 5:**
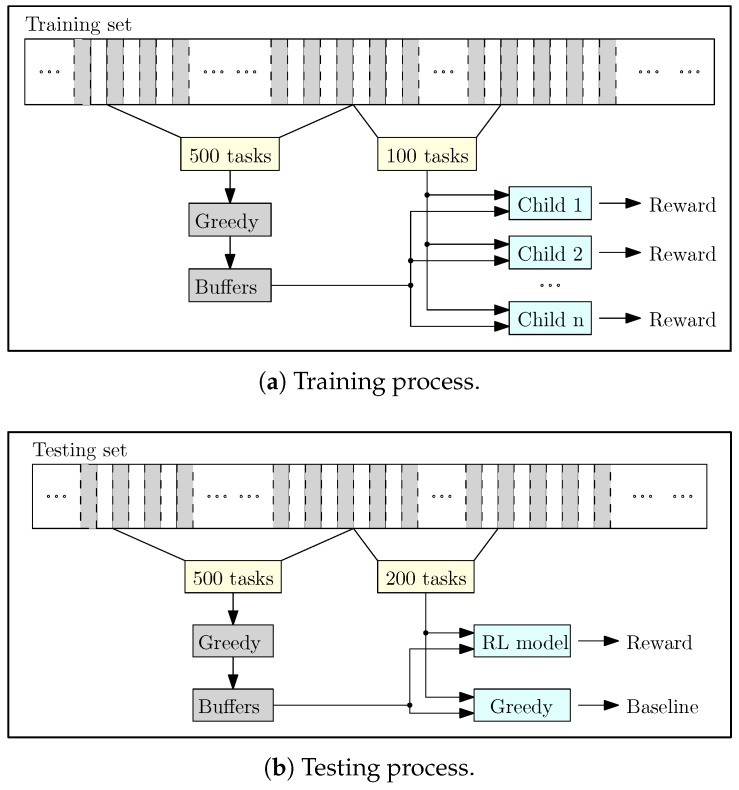
Training and testing process in the experiments.

We conducted 11 independent experiments, which are, i.e., at the beginning of an experiment, buffers of the servers are empty, and the result of the experiment does not affect other experiments. From the task uploaded dataset in Section 5.1.1, we divide 1 million tasks for testing and the rest for training the RL model. (Since our objective to the real-time task assignment is to optimize long-term latency, when we mention *n* tasks, it implies *n* consecutive tasks). Each experiment includes 10,000 iterations, where each iteration includes training and testing phases. In the training phase (Figure 5a), 600 tasks are randomly chosen for each iteration. The first 500 tasks are included in the chosen tasks replayed and assigned to the servers using the greedy method. (When *n* consecutive tasks are replayed, they exactly follow the uploading time, i.e., the time spans between task uploading times do not change). The reason we apply the greedy method to the first 500 tasks is to make sure that the buffers are in a normal status. If the buffers are empty, the latency of the system is low and it does not exactly express long-term latency of the system. We store the status of the buffers at the moment right before the task 501 is uploaded (which is the first task of the next 100 consecutive tasks).

We generate 10 children of the model following the method in Section 4. For each child, the initial state is a combination of the stored buffer and the first task in the 100 consecutive tasks, which craft a state as mentioned in Section 3.2. With each task assignment, the child receives a reward (which is the inverse of the latency at that moment). The average reward after 100 task assignments is the reward of the child. After all the children receive rewards, we update the system following the Algorithm 1. As a consequence of the training, the RL model maximizes the reward of the system over every 100 consecutive tasks.

In the testing phase, we randomly choose 700 consecutive tasks in the testing set. Task assignment method for the first 500 tasks is greedy, which is similar to the training phase. However, for the next 200 tasks, for each task, we observe the state of the system, and the trained RL model chooses a server for task assignment based on the state. The average reward over 200 task assignments is the reward of the testing (Figure 5b). We repeat the testing five times and the average reward over five times is the reward of the system after the iteration. Each time the reward of the system after an iteration is higher than the maximum reward of the system in the previous iterations, we store the weight matrices of the RL model and discard the previously stored matrices. After 10,000 iterations, the RL model with the stored weight matrices expresses the model that gives the maximum reward (Algorithm 1).

The rewards given by the testing are compared to the greedy algorithm. The greedy-based result is calculated as follows. An average reward is received after a process that is similar to testing; however, we apply the greedy method to the last 200 tasks (Figure 5b). The process is repeated 100 times and the average reward over 100 times is the reward of the system with the greedy method.

### 5.3. Results

Table 3 is a summary of the parameters utilized in the experiments. The middle column lists all possible values of a parameter, whereas the role of the value in the last column is not only the initial value of the parameter but also an anchor when we explore the effectiveness of other parameters. For instance, when the number of IoT devices changes in a range, the number of Fog servers is set to 5. In the case where the initial value of a parameter is fixed, the parameter does not change its value throughout the experiments. A fixed value of a parameter is the optimal value that makes the best contribution to the results and is discovered by grid search.

Based on the values of parameters in Table 3, 11 experiments were conducted. A summary of the results of the experiments is listed in Table 4. In each part of the table, values corresponding to a parameter are highlighted to indicate that we are changing the value of the parameter to discover its effect on the final results. For this reason, other parameters are set to initial values. The only exception is the number of IoT devices and the number of fog servers. Since the parameters are important in the design of the system, we explore their roles with two different experiments.

**Figure 6:**
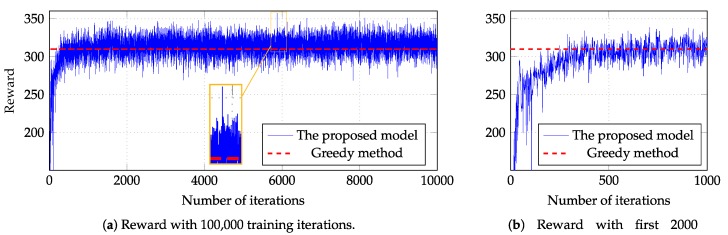
Reward with 100 IoT devices and five fog servers.

In experiment 1, the number of fog servers and IoT devices are set to five and 100, respectively. We scrutinize the experiment by plotting the result of 10,000 iterations as shown in Figure 6a. In this figure, the thin blue plot denotes the rewards in all iterations; the dashed red line is the greedy-based results, i.e., the reward when we apply greedy method. Figure 6b has the same presentation but the results of the first 1000 iterations only are shown. In the experiment, the proposed model overcomes the greedy method after a few iterations more than the first 200 iterations and peaks near the 60,000-th iteration (a magnified area). More specifically, Table 4 indicates that the proposed model outperforms the greedy method by 15.309%.

In experiment 2, the number of IoT devices increases to 200. Thereby, the average task uploading rate is 19.95 Gigacycles per second. To maintain the ratio of the average task uploading rate and the total capability of the servers at 0.96 (as in experiment 1), we increase the total capability to 20.8 GHz. The number of servers in this experiment is 10, and the frequency of their processors’ servers ranges from 1.56 GHz to 2.47 GHz. The improvement in experiment 2 is 16.101%, which is much higher than the improvement in experiment 1. The reason for the significant increase in the improvement with 10 servers, is that the RL model has more options for assigning the tasks to be uploaded, i.e., the model can find the better solution for the task assignment problem in general. However, an increase in the number of fog servers impacts the training time of the model. The reason is that the model has to calculate a probability that a server is chosen for task assignment among 10 servers, which takes more time than choosing a server among five servers in experiment 1. More specifically, average runtimes per iteration of experiments 2 and 1 are 1.667 s and 1.496 s, respectively, i.e., tasks in experiment 2 require 11% more time for each iteration than those in experiment 1.

**Figure 7:**
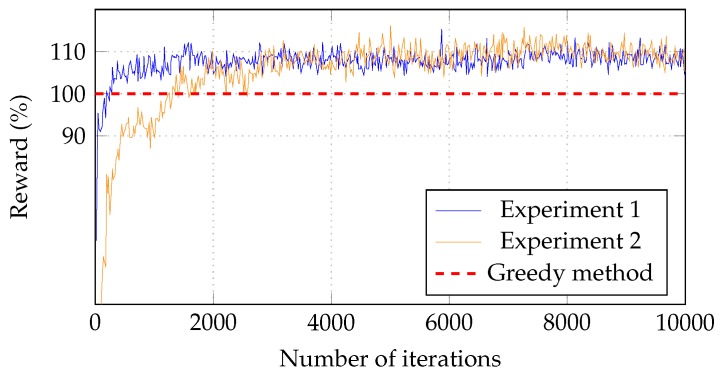
Exploring efficiency with number of fog servers.

Figure 7 illustrates a comparison of results in experiments 1 (exp1) and 2 (exp2). In the second experiment, the RL needs more time to reach the greedy-based results than in experiment 1. In fact, the model only reaches the greedy-based result after 1000-th iteration, which is five times compared to the 200-th iteration in the model in experiment 2. The result expresses that the complexity of the problem of task assignment in fog computing significantly increases in the case when the number of IoT devices increases. Thereby, the number of fog servers and the total capability of a server correspondingly increase. Consequently, the RL model takes more time to find the solution to the problem. On the other hand, the RL in experiment 2 has an optimal solution to the problem after 5000-th iteration, which is not much different than experiment 1 that has the optimal solution after 6000-th iteration. In other words, the RL in experiment 2 incurs a burden with an increase in the number of IoT devices in the system; however, it has the ability to find the optimal solution for the task assignment problem as well. From the experiments, it can be confidently shown that the RL approach works well with various fog computing systems.

**Figure 8:**
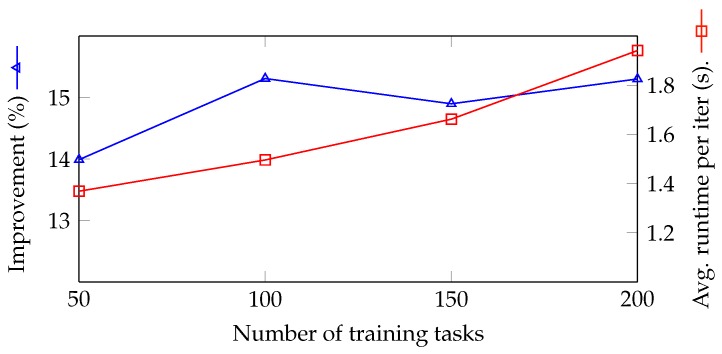
Exploring efficiency with a number of training tasks.

We explore the effectiveness of the number of training tasks in Figure 8. The blue line indicates the improvement of the proposed model in the experiments 3, 1, 4, and 5 compared to the greedy-based result, and the red line is the average run time per iteration of the experiments. The number of training tasks *n* indicates the number of consecutive tasks that the RL model needs to find the optimal solution (for task assignments). In other words, long-term latency optimization implies optimization in *n* consecutive tasks. Therefore, in case the number of training tasks is large, the RL model can optimize better. In contrast, the model may take more time to find the optimal solution, and in the worst case, it cannot find the optimal solution with 10,000 iterations. Figure 8 expresses the analysis, in which the result does not improve in case the number of training tasks is over 100, although the training time increases significantly. Conclusively, this parameter should be set at 100 for attaining the best result in the task assignment problem.

**Figure 9:**
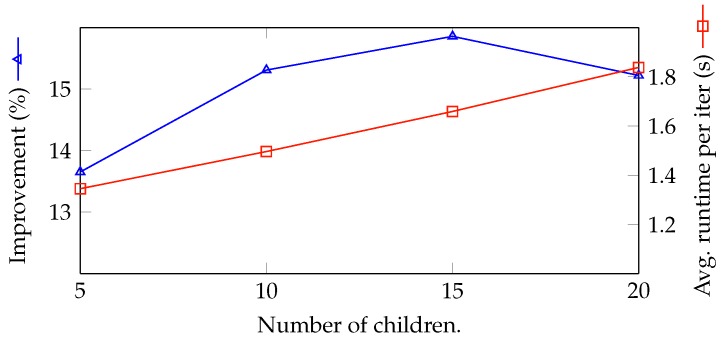
Exploring efficiency with number of children.

The effectiveness of the number of children generated in each iteration by the ES algorithm is explored in Figure 9. It is apparent that the runtime linearly grows corresponding to the number of children, since for each child, the RL has to run the same training and testing procedure. On the other hand, the improvements of the RL model peak for the population of 15 and decrease in the case of a larger population. Since the ES algorithm explores a solution in a nearby area, it needs enough samples to make a move toward the optimal area. This is the reason why the improvement grows corresponding to the number of children in the range of five to 15. On the other hand, too many samples do not help to improve the results, since it increases the risk of the NN getting stuck in the local optima, i.e., the model cannot find the best solution to the problem.

**Figure 10:**
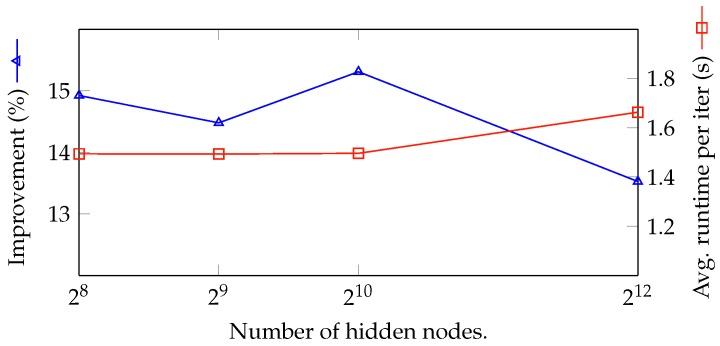
Exploring efficiency with the number of hidden nodes in NN.

In Figure 10, we explore the effectiveness of the number of hidden nodes in the NN to the final result. Since a neural network is a universal approximation function, an NN with more nodes approximates a function better than an NN with fewer nodes. Moreover, if the number of nodes in the NN is too small, the result is not stable enough. However, if the number of nodes is large, it takes much more time to train the network, and in the worst case, the NN cannot reach the optimal solution after 10,000 iterations. The figure clearly expresses the aforementioned analysis. The result fluctuates if the number of nodes is low. The network peaks at 1024 nodes and performs poorly in case the number of nodes is too large. It is worth noting that the number of hidden nodes only slightly affects the running time. In fact, if the number of hidden nodes in the NN is low, it does not affect the running time at all.

## 6. Conclusions

The long-term latency optimization of real-time task assignment is one of the most critical problems in the fog computing. The problem is difficult due to its high complexity; therefore, conventional optimization techniques do not work well. In this study, we resolve the problem with an RL model and apply the ES algorithm to optimize the model. The experiments show that the proposed model overcomes the greedy approach approximately 16.1% in terms of long-term latency for task execution. Moreover, the ES algorithm avoids the incorrect convergence to local optima which appears in most of the existing optimization methods based on the gradient. Additionally, the algorithm is embarrassingly parallel in implementation. Hence, it can speed up the learning process, particularly in practical frameworks and applications such as Omega, Mesos, Kubernetes, and Aneka [37,38,39,40]. To the best of our knowledge, the algorithm is applied to fog computing for the first time. This study also opens a new direction of the real-time task assignment in fog computing based on the RL and neuroevolution approach.

To extend our study, future works should consider an implementation of the proposed approach to real-world data sets such as telehealth big data and smart city on a testbed system. Another possible extension of this study is to use ES algorithm for simultaneously optimizing multiple utilities of the system in order to provide a balance between latency and energy consumption [10,11]. In addition, transmission latency when uploading task to fog servers or responding from servers to IoT devices should be taken into account.

## Figures and Tables

**Table 1:** Explanation of terms in the study.

Term	Explanation	Unit
Fog(*n*)	*n*-th fog server	
Exp(*n*)	*n*-th experiment	
Capability (i.e., frequency) of Fog(*n*)	Number of cycles that Fog(*n*) can complete per second	Hz
Size of a task	Number of bits in a task	bit
Complexity of a task	Number of cycles needed to solve a bit of the task	cycles/bit
Remaining tasks in a buffer	Tasks in the buffer at a given time	
Latency of Fog(*n*)	Computational latency of Fog(*n*) for completing the remaining tasks in its buffer	second
System latency	Maximum latency among all fog servers	second

**Table 2:** An example of real-time task assignment.

(**a**) **Greedy methods.**							
Time	Task	Size (Mbits)	Complexity (Cycles/bit)	Expected latency (Fog1-Fog2)	Fog1 latency	Fog2 latency	System latency
0 ms					0	0	0
	Task1	1	10	5 ms–10 ms	5 ms	0	5 ms
2 ms					3 ms	0	
	Task2	1	7	3.5 ms–7 ms	6.5 ms	0	
	Task3	1	8	4 ms–8 ms	6.5 ms	8 ms	8 ms
(**b**) **Long-term latency optimization.**							
Time	Task	Size (Mbits)	Complexity (Cycles/bit)	Expected latency (Fog1-Fog2)	Fog1 latency	Fog2 latency	System latency
0 ms					0	0	0
	Task1	1	10	5 ms–10 ms	5 ms	0	5 ms
2 ms					3 ms	0	
	Task2	1	7	3.5 ms–7 ms	3 ms	7 ms	
	Task3	1	8	4 ms–8 ms	7 ms	7 ms	7 ms

**Table 3:** Simulation parameters.

Parameter	Experiment	Initial
# of Fog servers	5, 10	5
# of IoT nodes	100, 200	100
# of training tasks	50, 100, 150, 200	100
Learning rate	0.002	Fixed
# of children	5, 10, 15, 20	10
Deviation of children	0.2	Fixed
# of hidden nodes in NN	256, 512, 1024, 4096	1024

**Table 4:** Experiments.

Experiment	# of Servers	# of IoT Devices	# of Training Tasks	# of Children	# of Hidden Nodes in NN	Our Model	Greedy Method	Improvement (%)	Average Runtime Per Iter (s)
1	**5**	**100**	100	10	1024	357.305	309.867	15.309	1.496
2	**10**	**200**	100	10	1024	346.509	298.455	16.101	1.667
3	5	100	**50**	10	1024	353.208	309.867	13.989	1.369
1	5	100	**100**	10	1024	357.305	309.867	15.309	1.496
4	5	100	**150**	10	1024	356.030	309.867	14.898	1.662
5	5	100	**200**	10	1024	357.2822	309.867	15.302	1.941
6	5	100	100	**5**	1024	352.169	309.867	13.652	1.345
1	5	100	100	**10**	1024	357.305	309.867	15.309	1.496
7	5	100	100	**15**	1024	359.002	309.867	15.857	1.659
8	5	100	100	**20**	1024	357.031	309.867	15.221	1.838
9	5	100	100	10	**256**	356.107	309.867	14.923	1.494
10	5	100	100	10	**512**	354.732	309.867	14.479	1.493
1	5	100	100	10	**1024**	357.305	309.867	15.309	1.496
11	5	100	100	10	**4096**	351.781	309.867	13.526	1.663

## References

[1] Mouradian C., Naboulsi D., Yangui S., Glitho R.H., Morrow M.J., Polakos P.A. (2017). A Comprehensive Survey on Fog Computing: State-of-the-art and Research Challenges. IEEE Commun. Surv. Tutor..

[2] Abbas N., Zhang Y., Taherkordi A., Skeie T. (2018). Mobile edge computing: A survey. IEEE Internet Things J..

[3] Dao N.N., Vu D.N., Lee Y., Park M., Cho S. MAEC-X: DDoS prevention leveraging multi-access edge computing. Proceedings of the IEEE International Conference on Information Networking (ICOIN).

[4] Shih Y.Y., Chung W.H., Pang A.C., Chiu T.C., Wei H.Y. (2017). Enabling low-latency applications in fog-radio access networks. IEEE Netw..

[5] Rahman G.S., Peng M., Zhang K., Chen S. (2018). Radio Resource Allocation for Achieving Ultra-Low Latency in Fog Radio Access Networks. IEEE Access.

[6] Dao N.N., Lee Y., Cho S., Kim E., Chung K.S., Keum C. Multi-tier multi-access edge computing: The role for the fourth industrial revolution. Proceedings of the IEEE International Conference on Information and Communication Technology Convergence (ICTC).

[7] Peng M., Yan S., Zhang K., Wang C. (2016). Fog-computing-based radio access networks: Issues and challenges. IEEE Netw..

[8] Vu D.N., Dao N.N., Jang Y., Na W., Kwon Y.B., Kang H., Jung J.J., Cho S. (2018). Joint energy and latency optimization for upstream IoT offloading services in fog radio access networks. Trans. Emerg. Telecommun. Technol..

[9] Sutton R.S., Barto A.G. (1998). Reinforcement Learning: An Introduction.

[10] He Y., Yu F.R., Zhao N., Leung V.C., Yin H. (2017). Software-defined networks with mobile edge computing and caching for smart cities: A big data deep reinforcement learning approach. IEEE Commun. Mag..

[11] He Y., Zhao N., Yin H. (2018). Integrated Networking, Caching, and Computing for Connected Vehicles: A Deep Reinforcement Learning Approach. IEEE Trans. Veh. Technol..

[12] Michalewicz Z. (1996). Evolution strategies and other methods. Genetic Algorithms + Data Structures = Evolution Programs.

[13] Salimans T., Ho J., Chen X., Sutskever I. (2017). Evolution strategies as a scalable alternative to reinforcement learning. arXiv.

[14] Bishop C., Bishop C.M. (1995). Neural Networks for Pattern Recognition.

[15] Nasrabadi N.M. (2007). Pattern recognition and machine learning. J. Electron. Imaging.

[16] Mahmud R., Kotagiri R., Buyya R. (2018). Fog computing: A taxonomy, survey and future directions. Internet of Everything.

[17] Mukherjee M., Shu L., Wang D. (2018). Survey of Fog Computing: Fundamental, Network Applications, and Research Challenges. IEEE Commun. Surv. Tutor..

[18] Mach P., Becvar Z. (2017). Mobile edge computing: A survey on architecture and computation offloading. IEEE Commun. Surv. Tutor..

[19] Markakis E.K., Karras K., Sideris A., Alexiou G., Pallis E. (2017). Computing, Caching, and Communication at the Edge: The Cornerstone for Building a Versatile 5G Ecosystem. IEEE Commun. Mag..

[20] Kim S. (2018). 5G Network Communication, Caching, and Computing Algorithms Based on the Two-Tier Game Model. ETRI J..

[21] Paščinski U., Trnkoczy J., Stankovski V., Cigale M., Gec S. (2018). QoS-aware orchestration of network intensive software utilities within software defined data centres. J. Grid Comput..

[22] Hu Y., Wang J., Zhou H., Martin P., Taal A., de Laat C., Zhao Z. Deadline-aware deployment for time critical applications in clouds. Proceedings of the European Conference on Parallel Processing.

[23] Chamola V., Tham C.K., Chalapathi G.S. Latency aware mobile task assignment and load balancing for edge cloudlets. Proceedings of the IEEE International Conference on Pervasive Computing and Communications Workshops (PerCom Workshops).

[24] Dao N.N., Vu D.N., Lee Y., Cho S., Cho C., Kim H. (2018). Pattern-Identified Online Task Scheduling in Multitier Edge Computing for Industrial IoT Services. Mob. Inf. Syst..

[25] Ali M., Riaz N., Ashraf M.I., Qaisar S., Naeem M. (2018). Joint Cloudlet Selection and Latency Minimization in Fog Networks. IEEE Trans. Ind. Inform..

[26] Dao N.N., Lee J., Vu D.N., Paek J., Kim J., Cho S., Chung K.S., Keum C. (2017). Adaptive resource balancing for serviceability maximization in fog radio access networks. IEEE Access.

[27] Witten I.H., Frank E., Hall M.A., Pal C.J. (2016). Data Mining: Practical Machine Learning Tools and Techniques.

[28] Mnih V., Kavukcuoglu K., Silver D., Rusu A.A., Veness J., Bellemare M.G., Graves A., Riedmiller M., Fidjeland A.K., Ostrovski G. (2015). Human-level control through deep reinforcement learning. Nature.

[29] Van Hasselt H., Guez A., Silver D. Deep Reinforcement Learning with Double Q-Learning. Proceedings of the AAAI.

[30] Wang Z., Schaul T., Hessel M., Hasselt H., Lanctot M., Freitas N. Dueling Network Architectures for Deep Reinforcement Learning. Proceedings of the International Conference on Machine Learning.

[31] Hornik K., Stinchcombe M., White H. (1989). Multilayer feedforward networks are universal approximators. Neural Netw..

[32] Silver D., Huang A., Maddison C.J., Guez A., Sifre L., Van Den Driessche G., Schrittwieser J., Antonoglou I., Panneershelvam V., Lanctot M. (2016). Mastering the game of Go with deep neural networks and tree search. Nature.

[33] LeCun Y., Bengio Y., Hinton G. (2015). Deep learning. Nature.

[34] Goodfellow I., Bengio Y., Courville A., Bengio Y. (2016). Deep Learning.

[35] Such F.P., Madhavan V., Conti E., Lehman J., Stanley K.O., Clune J. (2017). Deep Neuroevolution: Genetic Algorithms Are a Competitive Alternative for Training Deep Neural Networks for Reinforcement Learning. arXiv.

[36] Mnih V., Kavukcuoglu K., Silver D., Graves A., Antonoglou I., Wierstra D., Riedmiller M. (2013). Playing atari with deep reinforcement learning. arXiv.

[37] Schwarzkopf M., Konwinski A., Abd-El-Malek M., Wilkes J. Omega: Flexible, scalable schedulers for large compute clusters. Proceedings of the 8th ACM European Conference on Computer Systems.

[38] Hindman B., Konwinski A., Zaharia M., Ghodsi A., Joseph A.D., Katz R.H., Shenker S., Stoica I. Mesos: A Platform for Fine-Grained Resource Sharing in the Data Center. Proceedings of the 8th USENIX Conference on Networked Systems Design and Implementation.

[39] Bernstein D. (2014). Containers and cloud: From lxc to docker to kubernetes. IEEE Cloud Comput..

[40] Vecchiola C., Chu X., Buyya R. (2009). Aneka: A software platform for .NET-based cloud computing. High Speed Large Scale Sci. Comput..

